# Not All Fungi and Games: An Unusual Case of Fungal Keratitis in Michigan

**DOI:** 10.7759/cureus.92251

**Published:** 2025-09-13

**Authors:** Mary A Reiber, Lauren Touleyrou

**Affiliations:** 1 Infectious Disease, Michigan State University College of Human Medicine, Detroit, USA; 2 Infectious Disease, Detroit Medical Center/Wayne State University, Detroit, USA

**Keywords:** antifungals, cornea, fungus, keratitis, temperate climate

## Abstract

Fungal keratitis is a concerning cause of blindness that is significantly more common in tropical and subtropical areas of the world. Keratitis of fungal etiology has a more indolent course than bacterial keratitis due to many factors involving delayed presentation and misdiagnosis. Although fungal keratitis is more common in humid climates, Candida is the predominant species in temperate locations. Here, we report a case of fungal keratitis that developed in a 40-year-old female in Michigan, which was complicated by ulceration of the cornea.

## Introduction

Fungal infections cause a severe and diverse range of disease in humans and can be challenging to diagnose and treat [1]. Invasive fungal infections continue to affect a significant proportion of patients around the globe. It is estimated that fungi account for 20-60% of all culture-positive corneal infections in humid climates [2]. A study by Brown et al. published in 2021 investigates the global disease burden of fungal keratitis from 1946 to 2029 and estimated an annual incidence of approximately 1,050,000 cases per year, with the highest concentration of cases occurring in Asia and Africa, both of which are occupied by populations of groups in close proximity to the equator [2]. Fusarium species are reported to be the most common overall, but Aspergillus has been reported and Candida species are most common in temperate zones, although it is not clear why this is the case [2]. It might be due to predisposing factors in patients located in lower socioeconomic regions such as Asia or African countries who have less access to healthcare and may develop comorbidities that can cause immunosuppression (e.g. HIV/AIDS), and thus increase risk for opportunistic infections [2]. The higher incidence of fungal keratitis in tropical areas compared to temperate zones may contribute to the misdiagnosis of fungal keratitis for bacterial keratitis and subsequent failure of treatment. Additionally, more indolent courses of fungal keratitis can lead to patients presenting with more advanced cases, which further contributes to difficulty in treating these cases [1]. Patients may present with eye pain, photophobia, erythema, tearing and the sensation of a foreign body, which soon progresses to visual impairment and blindness [3]. Here, we present a case of fungal keratitis complicated by ulceration that occurred in the Midwest of the United States, a temperate zone, which failed the first round of treatment due to misdiagnosis, having been initially attributed to a bacterial etiology. Understanding the presentation of these infections, especially in areas outside of the tropical or subtropical areas can lead to improved surveillance and increase preservation of vision.

## Case presentation

A 40-year-old female, with no significant medical history, presented to a Michigan hospital in October 2021 with a two-week history of left eye pain with associated blurry vision and eyelid swelling. She had previously seen an ophthalmologist as an outpatient one week prior to admission, who prescribed her vancomycin and tobramycin eyedrops for treatment of bacterial keratitis. Due to lack of clinical improvement, she presented to the hospital. Patient denied any trauma to the eye and does not wear contact lenses.

**Figure 1:**
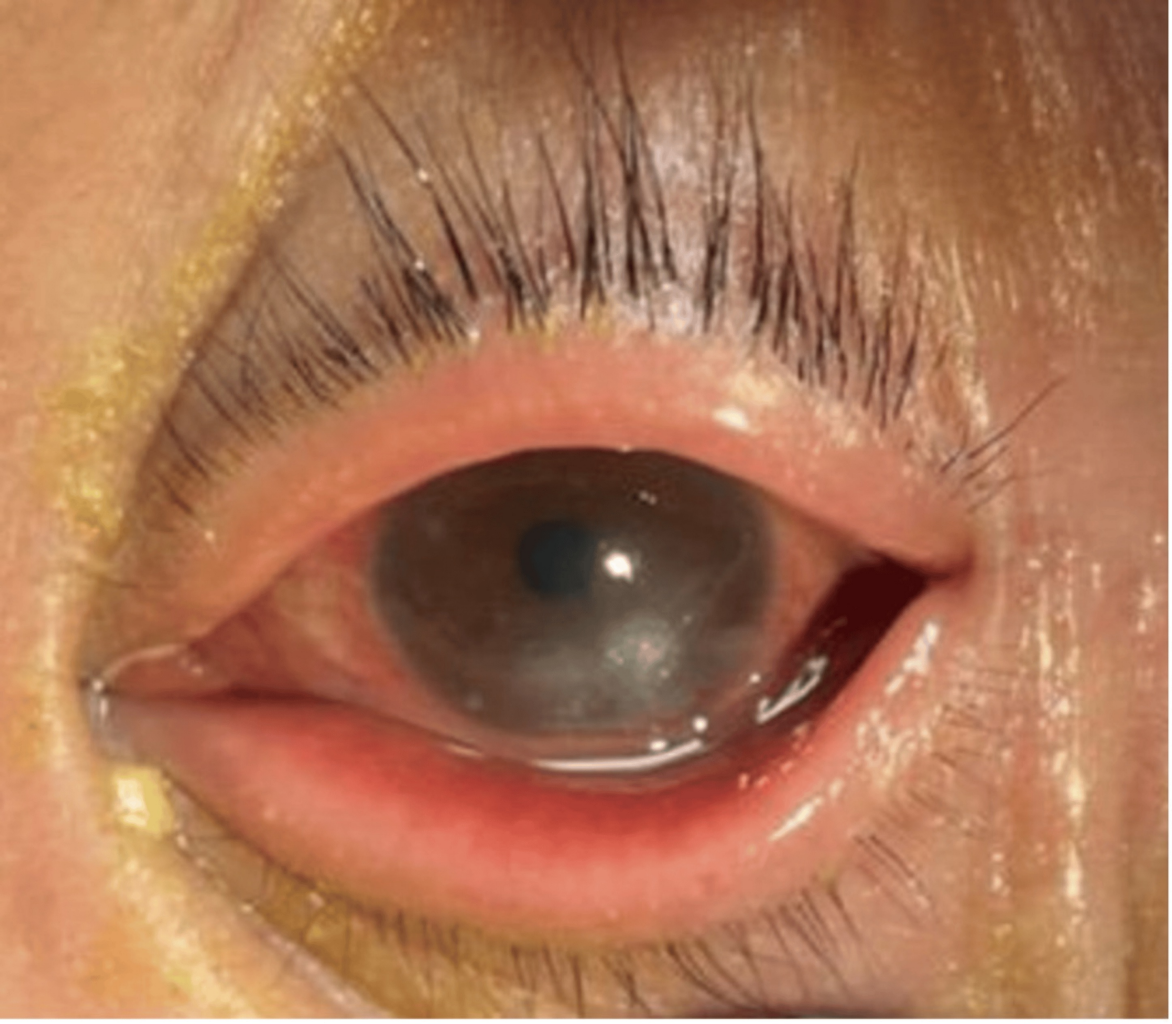
The patient’s left eye on admission. There is evidence of conjunctival erythema with purulent drainage and lower eyelid swelling.

**Figure 2:**
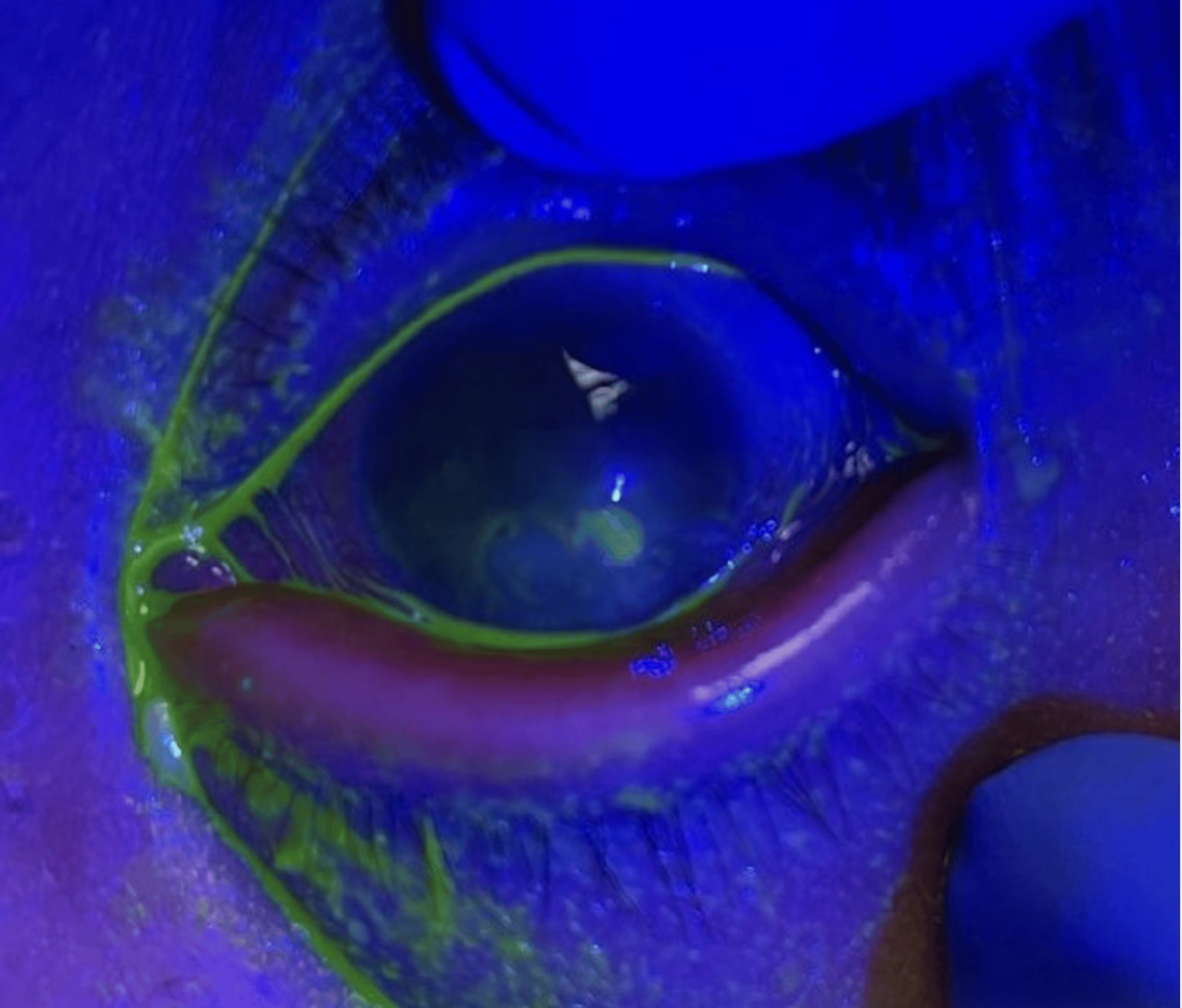
The infected eye with fluorescein dye. In the center of the cornea, is an ulcer with fluorescein uptake

On physical exam, patient appeared to be in mild distress due to the pain. She was hemodynamically stable and afebrile. Her eyelid appeared swollen with conjunctival erythema. She had yellow crusting around the eye and in the eyelashes (Figure 1). Her labs were within normal limits without evidence of inflammatory changes. Ophthalmologic evaluation of the patient revealed keratitis with infectious infiltrate. Fluorescein stain of the eye revealed a 2mm ulcer (Figure 2). 

**Figure 3:**
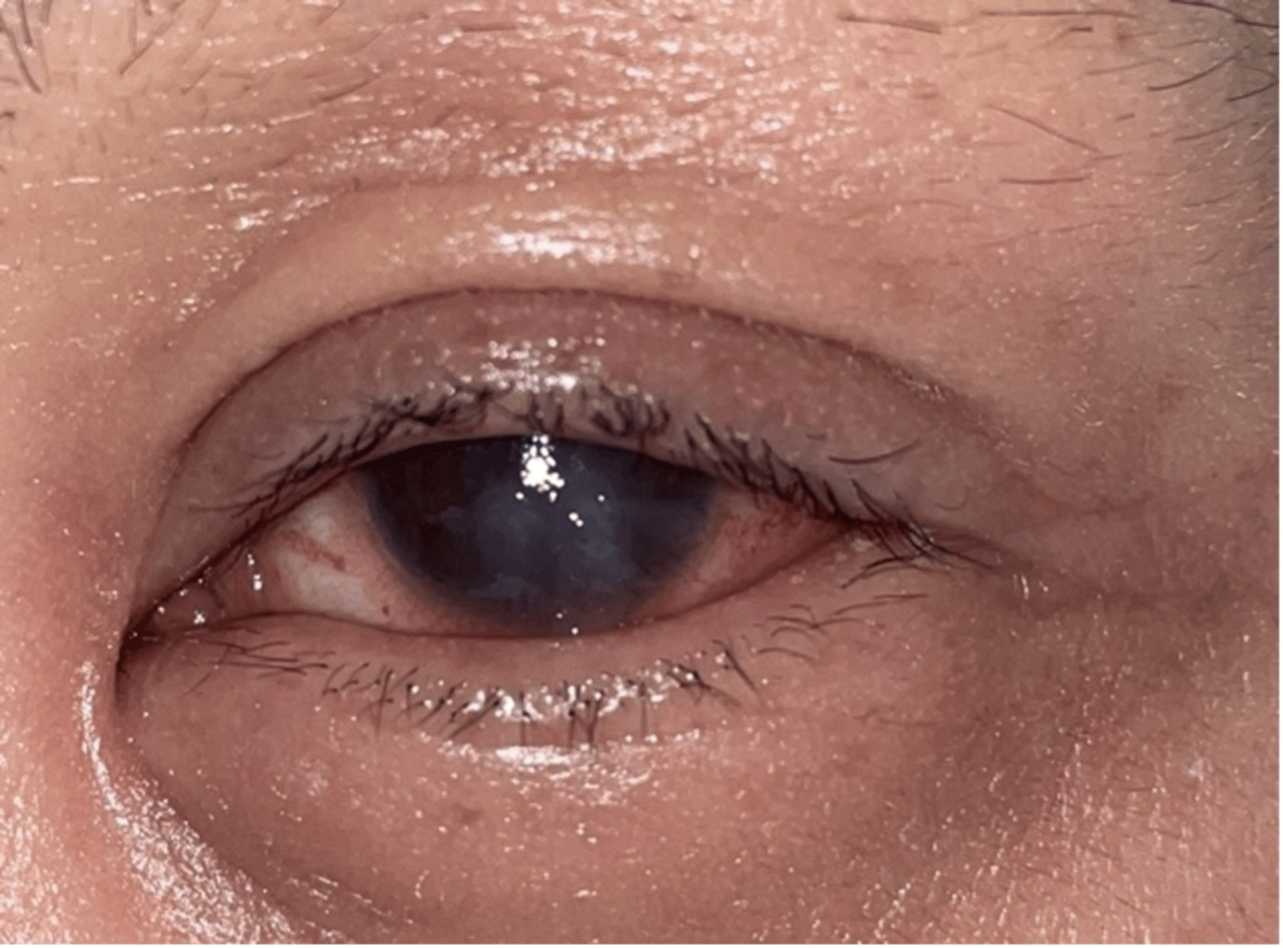
Post-op day three after receiving intrastromal voriconazole and amphotericin B

**Figure 4:**
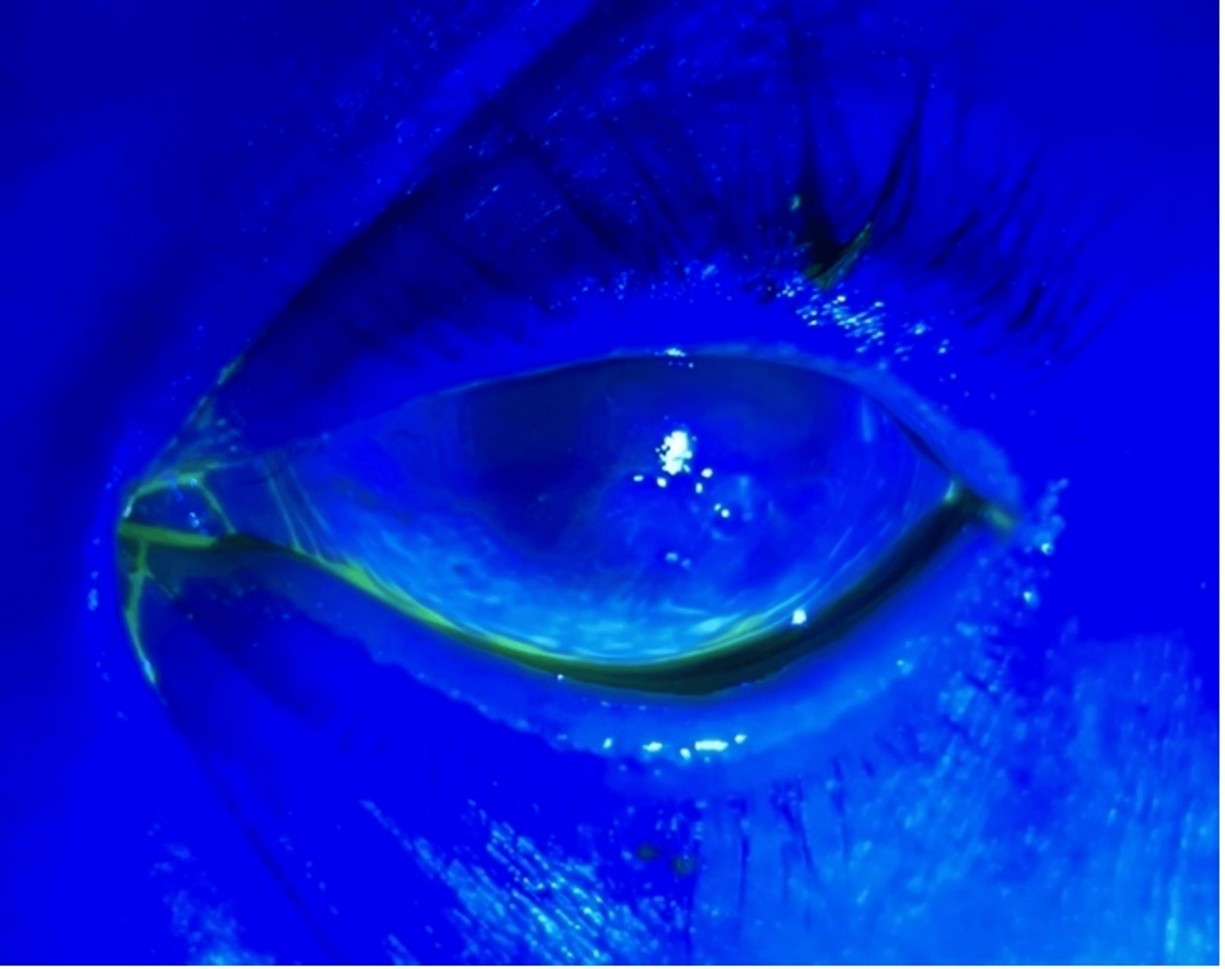
Fluorescein stain on post-op day three after receiving intrastromal voriconazole and amphotericin B

Corneal scraping of the eye revealed Candida albicans and Staphylococcus epidermidis. The patient was subsequently started on amphotericin B, voriconazole, moxifloxacin, and vancomycin eye drops every hour. She also underwent intrastromal voriconazole and amphotericin B injections on day two of hospitalization. She continued to show significant improvement and on day of discharge her eye showed minimal evidence of ulceration (Figures 3, 4). She was discharged on voriconazole eye drops with close ophthalmologic follow-up. The patient was noted to make great improvement in vision and swelling and was continued on topical therapy for two more weeks with almost complete resolution of vision.

## Discussion

Fungal keratitis has a much more significant disease burden in areas of lower socioeconomic status and areas with humid, warm climates, but still occurs in developed nations such as the United States which has an estimated incidence of six to eight cases per 100,000 annually [4]. While there are known to be 70 species of fungi that can cause fungal keratitis, the most common causes are Aspergillus, Fusarium, and Candida [4]. Candida is a dimorphic yeast and is known to be the more common etiology of general fungal ulcers in temperate climates [5]. Inciting events that can lead to this type of fungal keratitis could be trauma, especially microtrauma to the cornea, the use of contact lenses or topical steroid use.

Fungal keratitis tends to stay superficial and rarely causes endophthalmitis. When ulceration occurs in conjunction with a fungal keratitis infection, this indicates that the infection has progressed to its most severe form and is known as ulcerative fungal keratitis (UFK). Treatment of fungal keratitis is defined by the Mycotic Ulcer Treatment Trial (MUTT) I and II. MUTT I compared topical natamycin and topical voriconazole. The study found that topical natamycin treatment resulted in significantly better outcomes, notably for infections caused by Fusarium species [6]. However, there was no significant difference in outcome for patients with keratitis caused by other species of fungi. Based on this study, topical natamycin has become the preferred treatment [6]. However, depending on the species, voriconazole may provide similar or better results due to increased penetration and smaller molecule size of voriconazole [7]. MUTT II compared oral voriconazole and oral placebo. This study showed that topical therapy is non-inferior to systemic therapy and remains the mainstay of therapy and for fungal keratitis unless the ulcer is greater than 5mm with the depth of ulcer greater than 50% [8]. If patients show poor response after seven to 10 days of appropriate topical therapy, intrastromal antifungal injections are performed by ophthalmology. Additionally, if a patient is not responding to natamycin, topical amphotericin B could be administered; however, depending on the species of fungus causing the infection, this drug may not be effective [7]. Duration of therapy is determined on a patient-by-patient basis based on clinical improvement but generally is indicated for a longer period of time than bacterial keratitis because the response to topical antifungals tends to be slower than antibiotics [7]. Anti-fungal therapy can also be accompanied by cycloplegia treatment to reduce pain [7].

A description of the symptoms, interventions and outcomes of fungal keratitis is given in Table 1.

**Table 1:** Broad description of the symptoms, interventions and outcomes of fungal keratitis

	Symptoms	Interventions	Outcomes
Fungal Keratitis	Eye pain, redness and tearing, decreased vision with photophobia; Foreign body sensation; Visible corneal lesion or discharge	Topical Antifungals (Natamycin 5% or voriconazole 1%); Systemic antifungals for more severe infections; Targeted treatment via intrastromal injections	If detected early, resolution and treatment ;Possible recurrence or chronic infection; In severe cases may require surgical intervention such as penetrating keratoplasty or enucleation

## Conclusions

Fungal keratitis is a rarer form of infection of the cornea. Fungal species that can cause keratitis are present in warmer climates, and are known to have a decreased incidence in more temperate climates, such as the state of Michigan, which can make diagnosis and treatment more difficult for providers. Fungal keratitis is also less common than bacterial etiologies of keratitis, which further contributes to initial misdiagnosis and treatment delays. In patients who present with keratitis, it is vital to be aware of any microtrauma to the area, potential immunosuppression in the patient or contact use. Initiation of treatment for fungal keratitis should begin with topical antifungals, after culture or smear confirms a diagnosis and should continue for a longer course than other etiologies of keratitis in order to ensure proper treatment.
